# Duchenne Muscular Dystrophy from Brain to Muscle: The Role of Brain Dystrophin Isoforms in Motor Functions

**DOI:** 10.3390/jcm12175637

**Published:** 2023-08-29

**Authors:** Nalaka Wijekoon, Lakmal Gonawala, Pyara Ratnayake, Dhammika Amaratunga, Yetrib Hathout, Chandra Mohan, Harry W. M. Steinbusch, Ashwin Dalal, Eric P. Hoffman, K. Ranil D. de Silva

**Affiliations:** 1 Interdisciplinary Center for Innovation in Biotechnology and Neuroscience, Faculty of Medical Sciences, University of Sri Jayewardenepura, Nugegoda 10250, Sri Lanka; nalaka.wijekoon@gmail.com (N.W.); lak.gonawala@gmail.com (L.G.); 2 Department of Cellular and Translational Neuroscience, School for Mental Health and Neuroscience, Faculty of Health, Medicine & Life Sciences, Maastricht University, 6200 Maastricht, The Netherlands; h.steinbusch@maastrichtuniversity.nl; 3 Lady Ridgway Children’s Hospital, Colombo 00800, Sri Lanka; pyararatnayake959@gmail.com; 4 Princeton Data Analytics, Princeton, NJ 08544, USA; damaratung@yahoo.com; 5 School of Pharmacy and Pharmaceutical Sciences, Binghamton University, Binghamton, NY 13902, USA; yhathout@binghamton.edu (Y.H.); ehoffman@binghamton.edu (E.P.H.); 6 Department of Bioengineering, University of Houston, Houston, TX 77204, USA; cmohan@central.uh.edu; 7 Diagnostics Division, Center for DNA Fingerprinting and Diagnostics, Hyderabad 500039, India; ashwindalal@gmail.com; 8 Institute for Combinatorial Advanced Research and Education (KDU-CARE), General Sir John Kotelawala Defence University, Ratmalana 10390, Sri Lanka

**Keywords:** muscular dystrophy, DMD, natural history, South Asia, genotype–phenotype

## Abstract

Brain function and its effect on motor performance in Duchenne muscular dystrophy (DMD) is an emerging concept. The present study explored how cumulative dystrophin isoform loss, age, and a corticosteroid treatment affect DMD motor outcomes. A total of 133 genetically confirmed DMD patients from Sri Lanka were divided into two groups based on whether their shorter dystrophin isoforms (Dp140, Dp116, and Dp71) were affected: Group 1, containing patients with Dp140, Dp116, and Dp71 affected (n = 98), and Group 2, containing unaffected patients (n = 35). A subset of 52 patients (Group 1, n = 38; Group 2, n = 14) was followed for up to three follow-ups performed in an average of 28-month intervals. The effect of the cumulative loss of shorter dystrophin isoforms on the natural history of DMD was analyzed. A total of 74/133 (56%) patients encountered developmental delays, with 66/74 (89%) being in Group 1 and 8/74 (11%) being in Group 2 (*p* < 0.001). Motor developmental delays were predominant. The hip and knee muscular strength, according to the Medical Research Council (MRC) scale and the North Star Ambulatory Assessment (NSAA) activities, “standing on one leg R”, “standing on one leg L”, and “walk”, declined rapidly in Group 1 (*p* < 0.001 In the follow-up analysis, Group 1 patients became wheelchair-bound at a younger age than those of Group 2 (*p* = 0.004). DMD motor dysfunction is linked to *DMD* mutations that affect shorter dystrophin isoforms. When stratifying individuals for clinical trials, considering the *DMD* mutation site and its impact on a shorter dystrophin isoform is crucial.

## 1. Introduction

With an incidence rate of 4.80 per 100,000 [1], Duchenne muscular dystrophy (DMD, OMIM #310200), stands as the most prevalent form of muscular dystrophy in the pediatric population [2]. Thus far, a plethora of mutations in the *DMD* gene leading to the production of a truncated, nonfunctional dystrophin protein have been identified as the etiology of this X-linked recessive disorder [3].

**Figure 1:**
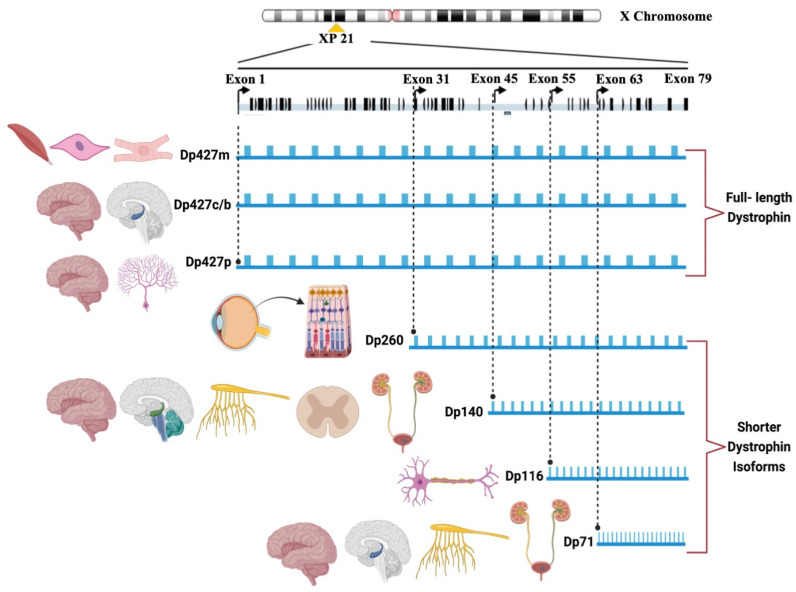
The DMD isoforms and their expression patterns.

The *DMD* gene is known to produce a minimum of seven major isoforms or variants of the dystrophin protein (Dp) through the utilization of seven established promoters [4]. From the upstream promoter/first exon sequences, three full-length isoforms are derived: Dp427m for muscle, Dp427c/b for cerebral, and Dp427p for Purkinje. Intriguingly, Dp427m and Dp427c are ubiquitously distributed across the cortex and basal ganglia in the human brain, with the hippocampus and amygdala exhibiting the highest expression levels and the cerebellum displaying the lowest. In addition, it is noteworthy that there exist four shorter isoforms, namely Dp260, Dp140, Dp116, and Dp71, with Dp140 and Dp71 being the most predominantly expressed in the brain. Conversely, the Dp260 and Dp116 isoforms are not expressed in the brain, with the former being exclusive to the retina and the latter exclusive to peripheral nerves [5,6,7] (Figure 1).

The significance of Dp140 and Dp71 in the involvement of the central nervous system (CNS) in DMD has been extensively studied [4,8,9,10,11,12] and was reviewed by Doorenweerd N et al., 2020 [13]. However, the relationship between brain function and motor performance in individuals with DMD is an emerging concept. In this context, Chesshyre et al., 2022, and Coratti et al., 2022, [12,14] reported that DMD boys lacking both the Dp140 and Dp71 isoforms experienced a notable decrease in their motor function as assessed by the North Star Ambulatory Assessment (NSAA), highlighting the cumulative effect of brain dystrophin isoform loss on motor performance. Further, the reported variations in motor function may be attributed to the direct influence of CNS involvement in motor executive function [12,14].

The objective of this study was to analyze the impact of multiple variables, including the cumulative loss of shorter dystrophin isoforms (Dp140, Dp71, and Dp116), age, and corticosteroid treatment, on the variability of motor outcomes in a group of 138 genetically confirmed DMD patients from a developing country, Sri Lanka, a geographically defined population in South Asia. To the best of our knowledge, the present study is the first of its kind from a South Asian perspective.

## 2. Materials and Methods

### 2.1. Patient Recruitment

Patient recruitment was conducted through neurology clinics in various government hospitals across Sri Lanka’s western, northwestern, north-central, central, southern, and northern provinces, as well as through pro bono mobile clinics and home visits.

A total of 138 male patients (age range (mean): 1.5–18 Yrs (8 Yrs)) genetically confirmed through multiplex ligation-dependent probe amplification (MLPA) (SALSA MLPA Kit P034/P035 DMD/Becker, MRC Holland, Amsterdam, the Netherlands) to have deletions/duplications in the *DMD* gene were enrolled in the study. The molecular diagnostic procedure was established by utilizing the primary molecular diagnostic recommendations, as outlined by Abbs et al. in 2010 [15], as well as the revised edition by Fratter et al. in 2020, in alignment with the European Molecular Quality Genetics Network’s (EMQN’s) optimal practice guidelines for genetic testing in dystrophinopathies [16].

The sociodemographic characteristics and clinical data of the patients were documented using a standard questionnaire and clinical batteries that included the NSAA, Vignos scale, Brooke scale, and Medical Research Council (MRC) scale for muscle strength. It is important to note that the clinical assessments, which included evaluating muscle strength using the MRC scale, were conducted by a medical practitioner by the pro bono research team under the guidance and observation of the principal investigator and the respective consultant neurologist or pediatric neurologist who was treating the patient. Information on developmental milestones was directly recorded from the child health development record (CHDR) of the Family Health Bureau, Ministry of Health Nutrition and Indigenous Medicine, Sri Lanka, during the baseline evaluation of the patient. Follow-ups were conducted for 52 patients, for which data from an extended 2nd follow-up were available for 20 patients, and follow-up data for up to the 3rd follow-up were available for 3 patients. The follow-ups were conducted at the following time intervals: the first follow-up occurred 24 months after the initial assessment, the second follow-up took place 48 months after the initial assessment, and the third follow-up was conducted 84 months after the initial assessment.

Every participant provided written informed consent where applicable. The assent of a proxy was obtained for patients unable to provide their own consent. This study adheres to the ethical standards of Sri Lankan institutional review boards that follow the Declaration of Helsinki (ethical approval Nos. 449/09 and 38/19 from the Ethics Review Committee, Faculty of Medical Sciences, University of Sri Jayewardenepura, and ethical approval No. LRH/D/06/2007 from the Lady Ridgeway Hospital for Children, Colombo 08, 0800, Sri Lanka).

### 2.2. Patient Categorization

Based on the mutation location and the published literature [4,5,10], missing dystrophin isoforms (Dp427m, Dp427c, Dp427p, Dp260, Dp140, Dp116, and Dp71) were predicted. The patients were categorized into the following two groups by considering whether single or multiple, shorter dystrophin isoforms expressed in the CNS (Dp140 and Dp71) and the peripheral nervous system (PNS) (Dp116) were affected or not. Group 1 comprised patients with single or multiple, shorter dystrophin isoforms expressed in the CNS and PNS that were affected (n = 98), and Group 2 comprised patients with shorter dystrophin isoforms expressed in the CNS and PNS that were not affected (n = 35).

### 2.3. Statistical Analysis

ANOVA tests and multivariate analysis were performed to analyze the effect of shorter dystrophin isoforms expressed in the CNS and PNS on the performance of patients in the motor function scales and their subsets. After conducting the exploratory data analysis, the impact of the averaged power variables (e.g., average ankle power) and the scores for NSAA subcategories across different age groups were examined while also considering the group effect. Regression models were fitted to analyze the relationship between the average power variable and age, as well as the NSAA subcategory scores and age, for Group 1 and Group 2, respectively. Scatterplots were used to visually compare the results. A Weibull regression analysis was performed using the follow-up data to identify the effect of shorter dystrophin isoforms expressed in the CNS and PNS on the age at which patients became wheelchair-bound, with the ages of the as-yet unaffected patients treated as censored observations. A statistical analysis was performed using R statistical software, version 4.2. Microsoft Power BI software was used for data visualization. Outlier analysis was performed statistically (Supplementary Figure S1), and 5 patients were identified as outliers and removed from further statistical analyses, leaving 133 patients for the final analysis (Table 1).

## 3. Results

### 3.1. Genotype and Demographic Characteristics of the Patient Cohort

**Figure 2:**
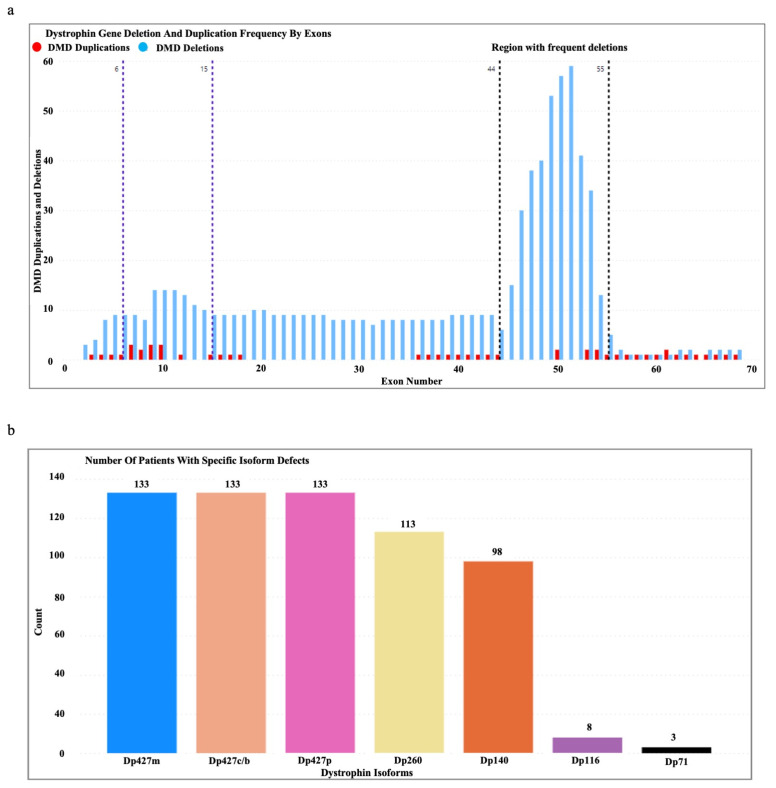
(**a**) Dystrophin gene deletion and duplication patterns in the patient cohort. (**b**) Number of patients with specific isoform defects (Dp427m, Dp427c, Dp427p, Dp260, Dp140, Dp116, and Dp71) identified based on the DMD gene mutation location.

A total of 133 male patients (age range (mean): 1.5–16 Yrs (8 Yrs)) genetically confirmed through MLPA to have deletions/duplications in the DMD gene were enrolled in the study. We observed the clustering of deletion mutations in exons 45–55 and regions 6–15 of the DMD gene and the clustering of duplications in exons 6–10 (Figure 2a). Almost all the patients had a defective Dp427m, Dp427c, and Dp427p, while mutations affecting the Dp116 and Dp71 isoforms were rare (Figure 2b).

The patients were categorized into the following two groups based on whether the shorter dystrophin isoforms expressed in the CNS (Dp140 and Dp71) and PNS (Dp116) were affected or not: Group 1 comprised patients with single or multiple, shorter dystrophin isoforms expressed in the CNS and PNS that were affected (n = 98), and Group 2 comprised patients with shorter dystrophin isoforms expressed in the CNS and PNS that were not affected (n = 35). Table 1 is a summary of the demographic characteristics of the patient cohort.

The distribution of patient ages and the ages of onset in each group exhibited a left skew, and no statistically significant differences in age (*p* = 0.4) or the age of onset (*p* = 0.6) between the groups were discovered. Interestingly, there was a significant difference (*p* = 0.02) in the age at which individuals in the two groups became wheelchair-bound. Specifically, patients in Group 1 (the median age upon becoming wheelchair-bound—9 years) lost their ability to walk at a younger age compared to those in Group 2 (the median age upon becoming wheelchair-bound—11 years) (Table 1).

When examining the relationship between age and creatine phosphokinase (CPK) values in patients, it was observed that there was a declining trend in the CPK values as age increased. However, there was no statistically significant difference observed in the decreasing trend of CPK values with age among the different groups (age of 0–5.5 Yrs, Group 1 vs. Group 2: *p* = 0.825; age of 5.5–9.75 Yrs, Group 1 vs. Group 2: *p* = 0.810; age of 9.75+ Yrs, Group 1 vs. Group 2: *p* = 0.981), as presented in Table 1.

The dosage of the corticosteroid (prednisolone) did not exhibit a significant difference (*p* = 0.19) between the patients in Group 1 and Group 2. It is important to mention that, during the baseline evaluation and follow-up periods, none of the patients were receiving corticosteroids. This is because the average age of the patients in both groups at the time of the baseline evaluation was 8 years, and corticosteroids were administered prior to the age of 6 years, as outlined in Table 1.

### 3.2. Effect of Shorter Dystrophin Isoforms Expressed in the CNS and PNS on the Performance in Motor Function Scales and Their Subsets

**Figure 3:**
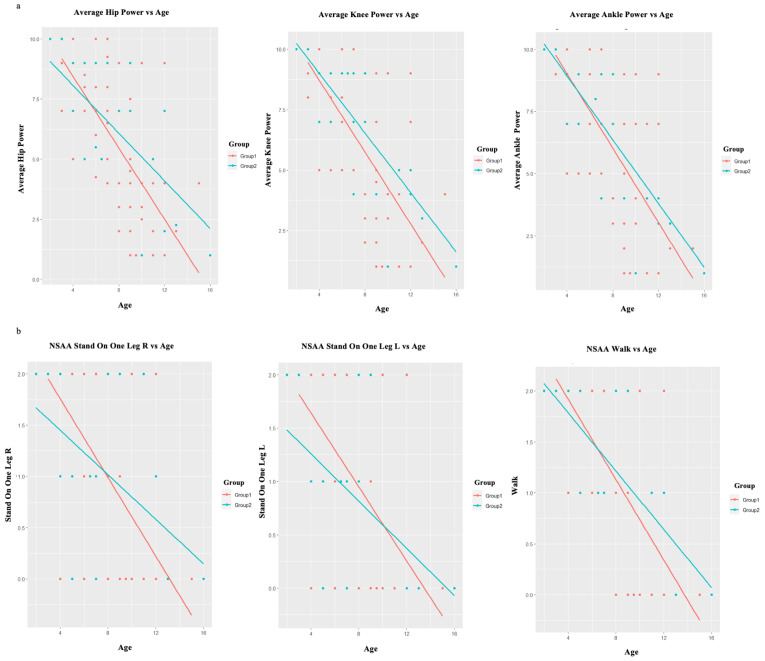
(**a**) The accelerated decline in hip muscle power and knee muscle power as measured by the MRC scale for muscle strength, with regard to age. (**b**) The accelerated decline in the performance of the activities of NSAA: “standing on one leg R”, “standing on one leg L”, and “walk” with regard to age. Group 1—red color, Group 2—blue color.

The study observed a significant difference (*p* < 0.001) in the deteriorating trend of the subcategories for the MRC scale and the NSAA concerning age between Group 1 and Group 2. This difference was evident in all subcategories among the patients in both Group 1 and Group 2, as shown in Table 2. Interestingly, an accelerated decline in hip muscle power (*p* < 0.001) and knee muscle power (*p* < 0.001) was observed concerning the MRC scale for muscle strength (Figure 3a). Despite the decline in muscle power associated with aging, there was no discernible accelerated decline observed in the upper-extremity muscles (Table 2). Moreover, the NSAA revealed that certain activities, namely “standing on one leg R” (*p* < 0.001), “standing on one leg L” (*p* < 0.001), and “walk” (*p* < 0.001), exhibited an accelerated decline in performance with age in Group 1 compared to Group 2 (Figure 3b and Table 2).

### 3.3. Follow-Up Evaluations of the Patients

**Figure 4:**
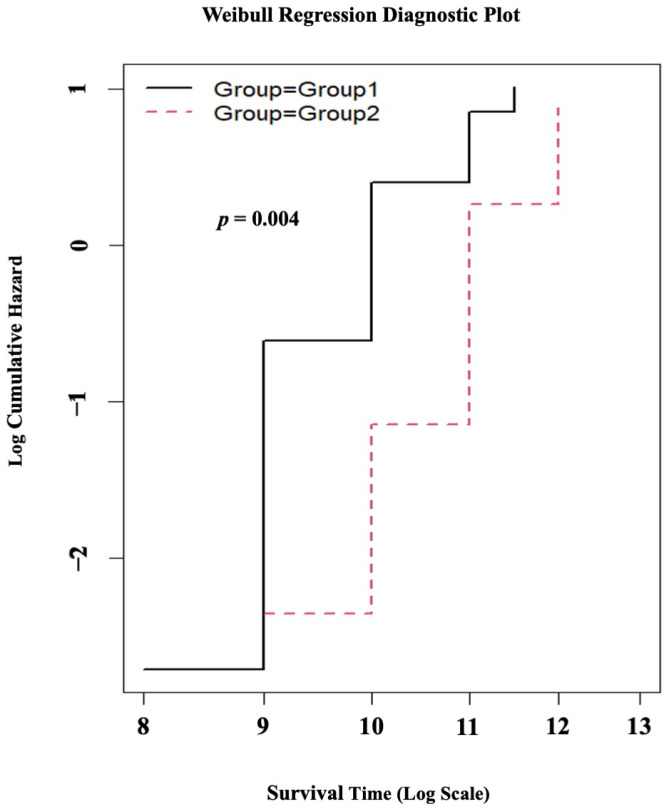
The results of the Weibull regression survival analysis. The black line represents Group 1, and the red dotted line represents Group 2.

The results of the Weibull regression survival analysis for the follow-up evaluations of the patients revealed a statistically significant difference (*p* = 0.004) for the age at which individuals became wheelchair-bound. Specifically, patients in Group 1 exhibited a tendency to become wheelchair-bound at a younger age with the progression of the disease compared to those in Group 2, as illustrated in Figure 4.

## 4. Discussion

The DMD gene variation hotspots of our cohort (Figure 2a) represent a distal variation hotspot spanning from exon 45 to 56, which is consistent with previous studies on DMD in South Asian populations [17,18,19,20]. While the investigation of the intricate correlation between shorter dystrophin isoforms and motor performance in DMD is a relatively new idea, previous research has examined the impact of brain dystrophin isoforms on the attainment of early developmental milestones [21,22,23,24,25]. These investigations have identified a delayed attainment of motor developmental milestones and language milestones in individuals with DMD. Nevertheless, it is important to acknowledge that there could potentially be delays in the attainment of milestones related to social, emotional, and behavioral development, as well as fine motor development [25].

Consistent with Dommelen et al., 2020, and as depicted in Table 1, a motor developmental delay was identified in our cohort as the most common type of developmental delay, while the least commonly observed issue was a delay in social–emotional or behavioral development. Importantly, there was a significant difference between Group 1 and Group 2 observed for the categories of developmental delays: motor developmental delays (*p* < 0.001), language delays (*p* < 0.001), and delays in vision and fine motor development (*p* = 0.038). These results suggest that the delay observed may be attributed to the involvement of dystrophin isoforms in the brain, specifically Dp140 and Dp71 isoforms, that have implications for brain functionality.

There has been an increasing emphasis in recent years on identifying early indicators of impairment in young boys affected with DMD [22,26,27,28,29]. In this context, Dommelen et al., 2020 [25], reported a correlation between developmental milestones and heightened susceptibility to DMD, where an inability to walk proficiently at 24 months was found to elevate the risk of DMD from 1 in 5000 to approximately 1 in 100 male infants. Despite the fact that 56% (74/133) of the patients in our cohort exhibited a delay in developmental milestones, only 35% (26/74) of these patients had their abnormality recognized by their parents at the onset, leading them to seek medical attention at an early age (Supplementary Table S1). If healthcare professionals and parents possessed adequate knowledge regarding the potential risk of DMD in male children exhibiting delayed developmental milestones, it is plausible that the age at which DMD is diagnosed could be decreased in 65% (48/74) of the remaining patients who had a delay in developmental milestones. Therefore, it is contended that commencing investigations on DMD with the identification of developmental delays could potentially lead to an earlier diagnosis.

Diagnostic delays in DMD remain frequent in traditionally disadvantaged groups, including patients from developing countries and those with a lower socioeconomic status [30,31]. Therefore, a potential approach could be suggested that involves identifying young male children with delayed developmental milestones and examining them for suggestive clinical symptoms of muscular dystrophy, followed by performing a CPK test. This would be a cost-effective approach to practice, even in a primary care setting in countries with limited resources, to achieve the early clinical detection of DMD. This approach could be further expanded when coupled with a cost-effective molecular diagnostic approach such as multiplex PCR followed by MLPA, which would facilitate the early confirmation of DMD.

One of the limitations observed in prior studies examining developmental delays in large cohorts of individuals with DMD [23,32] is the absence of developmental milestones documented by healthcare professionals and parental reporting in a retrospective context, which may be influenced by recall bias. To address this issue, Dommelen et al., 2020, examined the developmental achievements of infants with DMD during their initial months of life, with the medical professionals involved in the study being unaware of the participants’ diagnoses [25]. In our study, we sought to mitigate the concerns surrounding retrospective parental reporting and recall bias by implementing a method where the information about developmental milestones was directly recorded from the CHDR, as described in the methodology. The CHDR serves as a pivotal document within the National Growth Monitoring and Promotion Programme for children under the age of five in Sri Lanka.

Individuals with DMD experience a decline in their ability to walk independently, with a loss of independent ambulation occurring around the age of 13 years [33]. In our patient cohort, the median age at which individuals in Group 1 experienced wheelchair dependency was 9 years, whereas for Group 2, it was 11 years (*p* = 0.02) (Table 1). Furthermore, the survival analysis for the follow-up evaluation of the patients revealed that, with the progression of the disease, the patients in Group 1 were more prone to becoming wheelchair-bound at a younger age compared to those in Group 2 (*p* = 0.004), as depicted in Table 3 and Figure 4. The reported data on the age of wheelchair dependency in DMD patients of different populations are as follows: in India, the age is 10.4 years [18] and 13 years [34]; in Iran, the age is 10.9 years [35]; in Saudi Arabia, the age is 10.1 years [36]; in the Netherlands, the age is 9.7 years [37]; and in the UK, according to the North Star database and other related studies, the age is 13 years [10,38]. The variability observed in the age at which individuals become dependent on wheelchairs in different populations can potentially be attributed to regional disparities in patient management, healthcare resources, the socioeconomic background of the patients, genetic factors, and the influence of disease modifier genes specific to certain populations [30,39,40,41,42].

Additionally, it was postulated that mutations occurring in the DMD gene, which are expected to disrupt brain dystrophin isoforms, could potentially impact motor outcomes among individuals diagnosed with DMD [12,14]. Moreover, Cyrulnik et al., 2007, put forth the argument that DMD can be characterized as a “cerebellar disorder” due to the expression of the Dp140 and Dp71 brain dystrophin isoforms [5,23]. They proposed that the absence of dystrophin during brain development could potentially impair the effectiveness of the cerebro–cerebellar pathways. Interestingly, there are reports indicating that cerebro–cerebellar loops establish connections between the lateral regions of the cerebellar hemisphere and the cerebral cortex. These loops have conventionally been associated with the coordination of movement [43,44,45].

Taking into consideration the aforementioned, our study observed a significant difference (*p* < 0.001) in the decline pattern of the subcategories for the MRC scale and the NSAA in relation to age when comparing Group 1 and Group 2 (Table 2). It is noteworthy that a rapid deterioration in hip muscle power and knee muscle power was observed based on the MRC scale for muscle strength (Figure 3a). In a comparable manner, the NSAA demonstrated that certain activities, specifically “standing on one leg R”, “standing on one leg L”, and “walk”, displayed a heightened deterioration in performance with increasing age in relation to Group 1 to Group 2 (Figure 3b). It is important to highlight that, in contrast to the MRC scale, the NSAA is documented to possess a greater complexity and necessitate elevated levels of motor coordination and planning [14]. Hence, considering the role of Dp140 and Dp71 in brain function, it can be contended that our observations regarding the rapid decline of motor abilities in individuals with DMD may be associated with the impact of the CNS on motor executive functions [11,46].

It is plausible to hypothesize that the observed variability in the functional outcomes could be attributed to the variability in the expression of Dp71 in skeletal muscle. A recent study identified the expression of Dp71 in skeletal muscle [47], although it is widely reported that Dp71 is primarily expressed in the brain [48]. Furthermore, numerous studies have indicated the involvement of Dp71 in the proliferation of myoblast cells [49,50] and the activation of satellite cells [51]. These findings suggest that the lower expression of Dp71 in the muscles of individuals with DMD is a secondary event that is closely associated with the muscle degeneration and regeneration that are characteristic of DMD [14].

Furthermore, it is important to highlight that the administration of corticosteroids has the potential to exert an influence on the progression of DMD, as suggested by Zambon et al., 2022 [52]. In the present study, there was no statistically significant difference (*p* = 0.19) observed for the dosage of the corticosteroid (prednisolone) administered to patients belonging to Group 1 or Group 2, as presented in Table 1. It is imperative to note that, throughout the baseline assessment and subsequent follow-up periods, none of the patients were on corticosteroids. This is because the mean age of individuals in both cohorts during the initial assessment was 8 years, and the administration of corticosteroids occurred before the age of 6 years, as indicated in Table 1. Despite the fact that it is common practice to begin corticosteroids for DMD around the age of 4–5 years [53], an expert survey on the clinical practice of steroid usage in DMD performed in Asia and Oceania revealed that most clinicians in Asia and Oceania considered 4–7 years old to be an appropriate age to begin steroids for DMD [54]. This is consistent with the 5.6-year mean age of corticosteroid initiation in our patients (Table 1). Despite the fact that the most recent guidelines strongly advise continuing corticosteroids throughout life [55], the mean durations of corticosteroids in our group were 4.9 months and 4.4 months for individuals in Group 1 and Group 2, respectively. This was primarily because the parents of DMD children stopped using corticosteroids due to the adverse effects.

Our research will shed light on the growing emphasis on identifying early indicators of impairment in DMD, advising clinicians to identify young male children with delayed developmental milestones, examine them for suggestive clinical symptoms of muscular dystrophy, and then administer a CPK test to achieve the early clinical detection of DMD, even in a primary care setting. Furthermore, it is not standard practice in modern clinical trial settings to group patients based on the number of dystrophin isoforms affected. Our findings on the effect of the cumulative loss of brain dystrophin isoforms on the motor function outcome suggest that not stratifying clinical trial participants based on the cumulative loss of brain dystrophin isoforms may have a matrix effect on the outcome measurements, concealing the treatment effects. Moreover, we were able to identify 82/138 (60%) patients as amenable to the available exon-skipping therapies using MLPA diagnostics. Interestingly, the majority of our patients were eligible for exon 51 skipping (30/82), followed by exon 53 skipping (19/82) and exon 45 skipping (19/82). This was comprehensively discussed by the same authors in Wijekoon et al.’s 2023 study; thus, this is not discussed in this paper [56].

We would like to acknowledge the following limitations of this study. The evaluation of muscle strength using the MRC scale is reported to be evaluator-dependent. As mentioned in the methodology, the assessments were conducted by a medical practitioner to minimize this effect; however, we acknowledge the potential effect of evaluator dependency. Furthermore, because there are a limited number of patients with *DMD* mutations that specifically affect the Dp116 and Dp71 isoforms (Figure 2b), we had to combine patients with mutations in shorter dystrophin isoforms (Dp140, Dp116, and Dp71) into a single group in order to maintain statistical power. One limitation we acknowledge is that this approach limited our ability to distinguish the effects of each shorter dystrophin isoform on motor function. The analysis of the impact of dystrophin mutations on cardiac and respiratory phenotypes was hindered by the limited availability of data, which precluded the execution of a comprehensive statistical analysis. In addition, the annual follow-up evaluations were restricted to a cohort of 52 patients as a result of limitations imposed by the COVID-19 pandemic in Sri Lanka, which hindered clinic visits and home visits by the research team for a period of approximately two years.

## 5. Conclusions

In conclusion, we identified that mutations occurring in the *DMD* gene, which impact the expression of the shorter dystrophin isoforms Dp 140, Dp71, and Dp116, are correlated with the impairment of motor functions in individuals with DMD. Furthermore, it was evident that the cumulative loss of shorter dystrophin isoforms (Dp 140, Dp71, and Dp116) will have an effect on the natural history of DMD, where patients with mutations in the *DMD* gene affecting shorter dystrophin expression are more prone to having an accelerated decline in hip muscle power, knee muscle power, and NSAA activities involving the lower limbs. Moreover, it is advised for clinicians to identify young male children with delayed developmental milestones, examine them for suggestive clinical symptoms of muscular dystrophy, and then administer a CPK test to achieve early clinical detection of DMD, even in a primary care setting. Finally, it is suggested to consider the DMD mutation site and its effect on the cumulative loss of brain dystrophin isoforms when stratifying patients for clinical trials.

## Figures and Tables

**Table 1:** Summary of demographic factors for each patient group at baseline.

Details/Assessment	Baseline Assessment		*p*-Value
	Group 1	Group 2	
Number of patients	98	35	-
Median age (Yrs)	7	7	NS
Number of patients wheelchair-bound	18/98 (18%)	7/35 (20%)	-
Median age upon becoming wheelchair-bound (Yrs)	9	11	0.02
Minimum age upon becoming wheelchair-bound (Yrs)	8	9	NS
Median age of onset	4	4	NS
Developmental Delay			
Number of patients with any type of developmental delay	66	8	0.001
Motor developmental delay	59/66	8/8	0.001
Language delay	51/66	4/8	0.001
Vision and fine motor developmental delay	27/66	3/8	0.038
Clinical Assessments			
Average of NSAA	15	15	NS
Average of Brooke scale	2	1	NS
Average of Vignos scale	4	4	NS
Average of MRC scale total power	7	7	NS
Average CPK Levels for Different Age Groups			
Age of 0–5.5 Yrs	17,180.90	18,697.41	NS
Age of 5.5–9.75 Yrs	15,487.31	13,987.78	NS
Age of 9.75+ Yrs	7197.11	9116.60	NS
Details on Corticosteroid Usage			
Number of patients on corticosteroids	62/98 (63%)	28/35 (80%)	-
Mean age (Yrs) when corticosteroids were initiated	5.68 ± 0.95	5.64 ± 0.91	NS
Mean duration of the corticosteroid treatment (months)	5 months ± 1.39	4.46 months ± 1.26	NS
Mean dosage with a regime of 10 days on/off (mg)	Prednisolone 9.87 ± 4.17	Prednisolone 8.83 ± 3.56	NS
Number of corticosteroid-naïve patients	25/98 (26%)	6/35 (17%)	-
Mean age (Yrs) of corticosteroid-naïve patients	4.92 ± 1.15	4 ± 1.26	NS
Data not available on corticosteroid usage	11/98 (11%)	1/35 (2.8%)	-

NS—not significant.

**Table 2:** The deteriorating trend of the subcategories of the Medical Research Council (MRC) scale for muscle strength and the North Star Ambulatory Assessment (NSAA) with age in Group 1 (G1) and Group 2 (G2) at *p* < 0.001.

MRC Scale for Muscle Strength		
Muscle Group	Regression Coefficient G1	Regression Coefficient G2
Ankle	−0.748	−0.641
Hip	−0.744	−0.496
Knee	−0.593	−0.425
Elbow	−0.742	−0.617
Shoulder	−0.570	−0.419
Wrist	−0.551	−0.458
Fingers	−0.539	−0.437
Thumb	−0.552	−0.437
Neck	−0.192	−0.320
**North Star Ambulatory Assessment (NSAA)**		
**Subcategory/Activity**	**Regression Coefficient G1**	**Regression Coefficient G2**
Stand	−0.214	−0.175
Walk	−0.197	−0.143
Sit to Stand	−0.213	−0.165
Stand on One Leg R	−0.193	−0.109
Stand on One Leg L	−0.173	−0.111
Climb Steps R	−0.113	−0.118
Climb Steps L	−0.119	−0.088
Descend Steps R	−0.132	−0.123
Descend Steps L	−0.130	−0.103
Get to Sitting	−0.214	−0.175
Rise from Floor	−0.113	−0.100
Lift Head	−0.196	−0.195
Stand on Heels	−0.138	−0.111
Hop R	−0.123	−0.124
Hop L	−0.082	−0.135
Jump	−0.083	−0.135
Run	−0.121	−0.105

**Table 3:** Yearly follow-up data up to three follow-ups.

Details/Assessment	Follow-Up 1 *		Follow-Up 2		Follow-Up 3	
	Group 1	Group 2	Group 1	Group 2	Group 1	Group 2
Number of patients	38	14	15	5	2	1
Median age (Yrs)	9	11	11	15	13	20
Number of patients wheelchair-bound	14/38 (37%)	4/14 (29%)	12/15 (80%)	5/5 (100%)	2/2 (100%)	1/1 (100%)
Median age upon becoming wheelchair-bound (Yrs)	10	11	10	12	9	11
Minimum age upon becoming wheelchair-bound (Yrs)	8	10	8	11	9	11
Median age of onset	4	5	4	4	5	4
Average of NSAA	9	9	1	0	0	0
Average of Brooke scale	2	2	4	4	6	6
Average of Vignos scale	6	6	8	9	9	9
Average of MRC scale total power	6	6	4	3	2	2

* For both Group1 and Group 2, the follow-ups were performed at the following time intervals from the initial assessment: 1st follow-up: 24 months, 2nd follow-up: 48 months, and the 3rd follow-up: 84 months.

## Data Availability

The datasets used and/or analyzed during the current study are available from the corresponding author upon reasonable request.

## Supplementary Materials

The following supporting information can be downloaded at: https://www.mdpi.com/article/10.3390/jcm12175637/s1, Supplementary Figure S1: Outlier removal of the data set. Supplementary Table S1: Details of the abnormality identified by the parents at the onset, which prompted them to seek medical attention.
