# Supplementary material for: Duchenne Muscular Dystrophy from Brain to Muscle: The Role of Brain Dystrophin Isoforms in Motor Functions
Source: J Clin Med. 2023 Aug 29;12(17):5637. doi: 10.3390/jcm12175637 (PMC10488491; doi:10.3390/jcm12175637)
Supplement: Supplementary file 1 [file jcm-12-05637-s001.zip › Suppilimentaory Table S1.pdf]

Supplementary Table S1- Details of the abnormality identified by the parents at the onset, that prompted them to seek medical attention.

First neurological event	Group 01	Group 02
**Difficulty in climbing stairs**	36/98 (37%)	10/35 (29%)
**Frequent falling**	27/98 (28%)	15/35 (43%)
**Development delay**	23/98 (23%)	3/35 (9%)
**Difficulty in rising from floor**	4/98 (4%)	3/35 (9%)
**Calf hypertrophy**	3/98 (3%)	2/35 (6%)
**Toe walking**	3/98 (3%)	1/35 (3%)
**Calf Pain**	2/98 (2%)	0
