# Supplementary figures and images for: Duchenne Muscular Dystrophy from Brain to Muscle: The Role of Brain Dystrophin Isoforms in Motor Functions
Source: J Clin Med. 2023 Aug 29;12(17):5637. doi: 10.3390/jcm12175637 (PMC10488491; doi:10.3390/jcm12175637)

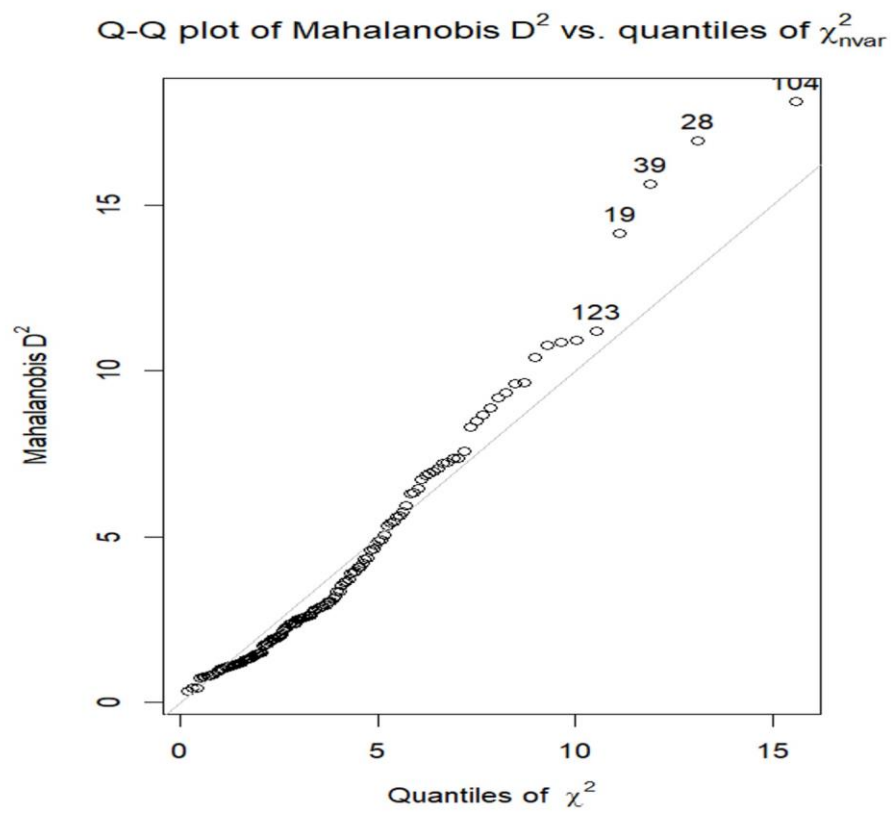

Supplementary Figure S1: Outlier removal of the data set.

Supplement: Supplementary file 1 [file jcm-12-05637-s001.zip › Supplimentary Figure S1.pdf]
